# Triple Test Screening for Down Syndrome: An Egyptian-Tailored Study

**DOI:** 10.1371/journal.pone.0110370

**Published:** 2014-10-20

**Authors:** Hazem S. Abou-Youssef, Manal M. Kamal, Dina A. Mehaney

**Affiliations:** Clinical and Chemical Pathology Department, Faculty of Medicine, Cairo University, Cairo, Egypt; NIDCR/NIH, United States of America

## Abstract

**Background:**

The incidence of Down syndrome (DS) in Egypt varies between 1∶555 and 1∶770 and its screening by triple test is becoming increasingly popular nowadays. Results, however, seem inaccurate due to the lack of Egyptian-specific information needed for risk calculation and a clear policy for programme implementation. Our study aimed at calculation and validation of the triple marker medians used in screening Egyptian females as well as to recommend programme conventions to unify screening in this country.

**Methods:**

The study was conducted on 668 Egyptian women, in weeks 15–20 of pregnancy as proven by sonar. Chorionic gonadotropin (CG), α-fetoprotein (AFP) and unconjugated oestriol (uE3) were measured on Siemens Immulite analyzer. Medians of the three parameters were calculated, regressed against gestational age (GA) and weighted by the number of participants/week. Equations were derived to adjust each parameter to the maternal weight and were centered on the median Egyptian weight. Prisca software was fed with the above data, multiples-of-median (MoM) and DS risks were calculated and the screening performance was evaluated at a mid-trimester risk cutoff of 1∶270.

**Results:**

Log-linear [AFP/uE3 = 10^(A+B^*^GA)^] and exponential equations [CG = A*e^ (B^*^GA)^] were derived and the regressed medians were found to follow similar patterns to other Asian and Western medians. Oestriol was always lowest (even halved) while CG and AFP were intermediate. A linear reciprocal model best fitted weight distribution among Egyptians and successfully adjusted each parameter to a weight of 78.2 kg. Epidemiological monitoring of these recommendations revealed satisfactory performance in terms of 6.7% initial positive rate and 1.00 grand MoM.

**Conclusions:**

Adoption of the above recommendations is hoped to pave the way to a successful DS screening programme tailored to Egyptian peculiarities.

## Introduction

Down syndrome (DS) is the most common chromosomal aneuploidy in live born infants. The overall incidence of DS is approximately 1 in 800 births in the general population [1]. Figures in Egypt vary between 1 in 555 in one study [2] to 1 in 770 in another [3]. In 2007, the American College of Obstetricians and Gynecologists (ACOG) recommended that all pregnant women, regardless of their age, should be offered screening for DS. Those with high risk should be confirmed by an invasive diagnostic procedure like amniocentesis or chorionic villus sampling [4].

Second trimester screening is traditionally based on the “triple test”. In this test, three maternal serum markers [α-fetoprotein (AFP), chorionic gonadotropin (CG) and unconjugated oestriol (uE3)] are measured and used to modify the women’s prior risk (based on her age) to yield a patient specific DS risk [5]. In order to compensate for variation of these markers with gestational age, the measured concentrations are divided by the median marker levels in the relevant gestational week yielding “Multiples of the Median (MoM)”. Furthermore, these MoMs are adjusted to compensate for factors, other than DS, that alter marker levels [6].

Most commercial software packages rely on the same algorithm (overlapping multivariate Gaussian distributions) for risk calculation. The quality of the results obtained depends therefore, among others, on the following variables: analytical performance of the immunoassays used [7]; accurate dating of pregnancy [8], proper choice of medians used to calculate the MoMs, and of valid factors for MoM adjustment and on the selection of suitable population parameters [9].

Despite the increasing number of triple test requests, risk calculations have not been tailored to the Egyptian peculiarities, leading to gross errors in risk prediction and a greater proportion of women unnecessarily exposed to invasive techniques. Aiming at bridging this serious gap in our practice, the current study was conducted.

Considering the economic situation of our region, adding a fourth test (quadruple testing) or combination with first trimester screening, though more efficient, was projected to decrease programme uptake. A wide-spread, less expensive but less efficient programme was thought to be more valuable to our community.

## Materials and Methods

The study was conducted on 668 pregnant women spanning gestational weeks 15^+0^ to 20^+6^. They were recruited from the Obstetric department of the Faculty of Medicine, Cairo University between December 2011 and November, 2012. Gestational dating for all women was based on biparietal diameter measurement, while dating based on last menstrual period (LMP) was only used to calculate the magnitude of error in dating and in subsequent risk calculation. Ultrasonography was also used to exclude multiple pregnancies and gross fetal anomalies. Blood samples were extracted from each participant and serum was separated and stored at −20°C for a period that did not exceed 6 days to ensure analyte stability [10]. All participants signed informed consent accepting all the procedures to be done. The study was approved by the Research Ethics Committee of The Faculty of Medicine, Cairo University. All specimens were analysed for AFP, CG and uE3 using Immulite 2000 analyser (Siemens Medical Solutions Diagnostics) and their corresponding reagents and calibrators; catalogue numbers: L2KAP2, L2KCG2 and L2KUE32, respectively). The technique is a solid-phase, enzyme-amplified chemiluminescence immunoassay that was run fully-automated according to the manufacturer instructions. Strict adherence to quality assurance procedures were followed throughout the study including acceptable performance of a two-level quality control material introduced in each run. DS risk was calculated using Siemens PRISCA Prenatal Risk Calculation Software v. 4.0, catalogue Number: 402692. Calculations were based on the population parameter set of Cuckle [11]. The used version of Prisca does not allow selecting between different parameter sets.

Medians values of the three parameters were calculated for each completed gestational week. These medians were smoothened by regression against the gestational weeks (average decimal weeks). A log-linear model best fitted the relation between GA and both AFP and uE3 whereas CG was described by an exponential equation. These regression equations were weighted based on the square root of the number of samples in each week.

To derive adjustment factors for maternal weight, a linear reciprocal model [12] was found to best fit the weight distribution of Egyptian women. An equation was derived to adjust the MoMs of each of the three parameters to maternal weight. Prisca default adjustment factors for insulin-treated diabetes, smoking and in-vitro fertilization were accepted. Since the observed medians were calculated from a homogeneous subset of Egyptians belonging to the same ethnic origin (Arab panethnicity) [13], no correction was warranted for ethnicity. A priori DS risk was calculated according to the formula of Snijders et al [14]. Data were statistically analysed using IBM SPSS v. 20.

## Results

The 668 studied females were fairly equally distributed among weeks 15 to 20, with no less than a hundred participants/week. Their ages at sampling time ranged between 15.3 and 47.7 years; median (interquartile range) were 27.8 (24.7–30.9) y with 93.3% younger than 35 y. Maternal weight ranged between 50 and 103 kg; median: 78.2 (76.3–82.6) kg. None of them declared smoking and only 1.6% were on insulin treatment. Raw data are presented in table S1.

**Figure 1:**
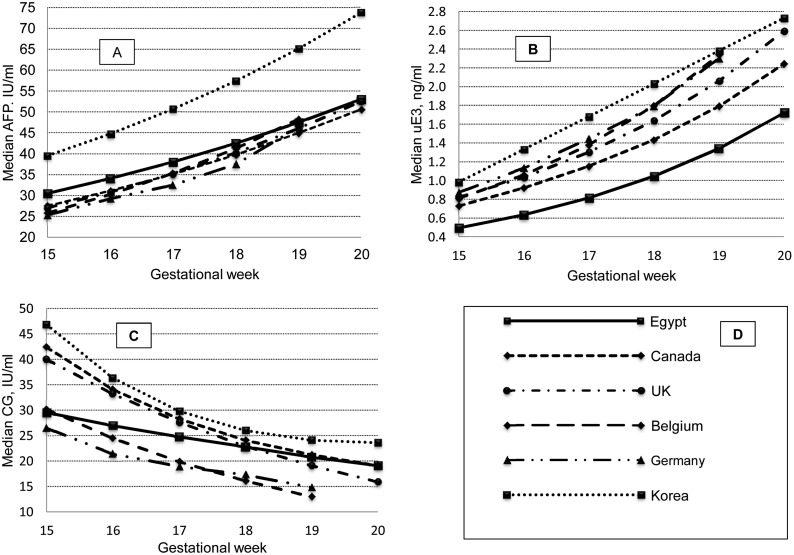
Comparison between median triple marker parameters in Egypt and different geographic regions. Panel A is AFP, B is uE3, C is CG and D is an explanatory legend.

The observed medians of the three parameters are given in table 1. When regressed against the average GA for each week gestational age, the equations describing the relation of median AFP and uE3 to gestational age were log-linear having the general layout: AFP (IU/ml) or uE3 (ng/ml) = 10^(A+B^*^GA)^. For CG, the equation was exponential; CG (mIU/ml) = A*e^(B^*^GA)^. These equations were weighted by the square root of the number of participants/week. Details of the equations are given in table 2. Using these median analyte values, the grand median MoM for each of the three parameters (without regard for the gestational week) was always 1.00. Figure 1 compares Egyptian medians with those in Korea [15] and a number of Western areas compiled by Vranken et al [16].

**Table 1:** Observed and regressed median values for the three measured triple marker parameters.

GA, wk	Mean GA	N	AFP, IU/ml		uE3, ng/ml		CG, mIU/ml	
			Obs	Reg	Obs	Reg	Obs	Reg
15	15.34	124	33.3	30.5	0.48	0.49	30 700	29 523
16	16.33	106	35.5	34.0	0.65	0.63	26 900	26 979
17	17.30	109	37.2	38.0	0.81	0.81	23 500	24 789
18	18.30	102	43.4	42.5	1.18	1.04	21 500	22 783
19	19.35	117	43.7	47.4	1.34	1.34	20 500	20 768
20	20.34	110	62.0	53.0	1.62	1.72	20 400	19 102

*GA,* gestational age in weeks; *N,* number; *AFP*, alpha fetoprotein; *uE3*, unconjugated oestriol; *CG*, chorionic gonadotropin, *Obs*, observed median, *Reg*, regressed weighted median.

**Table 2:** Regression coefficients for the equations relating the three measured triple marker parameters to gestational age (in decimal weeks).

	AFP, IU/ml	uE3, ng/ml	CG, mIU/ml
A (SE)^1^	0.759 (0.167)	−1.938 (0.036)	111899.7 (1950)
B (SE)^2^	0.048 (0.009)	0.109 (0.002)	−0.087 (0.003)
r (R^2^)^3^	0.928 (0.862)	0.990 (0.980)	0.984 (0.969)
P	0.007	<0.0005	<0.0005

1 Intercept (standard error);

2 Slope (standard error);

3 Correlation coefficient (coefficient of determination).

The linear reciprocal model for weight correction yielded the following general layout formula: *Weight-adjusted MoM* = 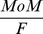; where F = 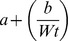, Wt is the maternal weight in kg, F for AFP = 
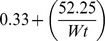
, for uE3 = 
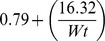
 and for CG = 
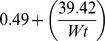
. Weight correction was centred on the median weight of the screened population (78.2 kg) and is applicable within the studied weight range (50 to 103 kg). Within this weight range, weight-correction factors ranged between 0.91–1.33 for AFP, 0.83–1.02 for uE3 and 0.93–1.25 for CG. Based on these correction factors, a woman’s MoM value may increase up to 120.5% or decrease by 75.2%; particularly at the extremes of weights studied. Such factors were constantly 1.00 for the three analytes for women weighing 78.2 kg.

**Figure 2:**
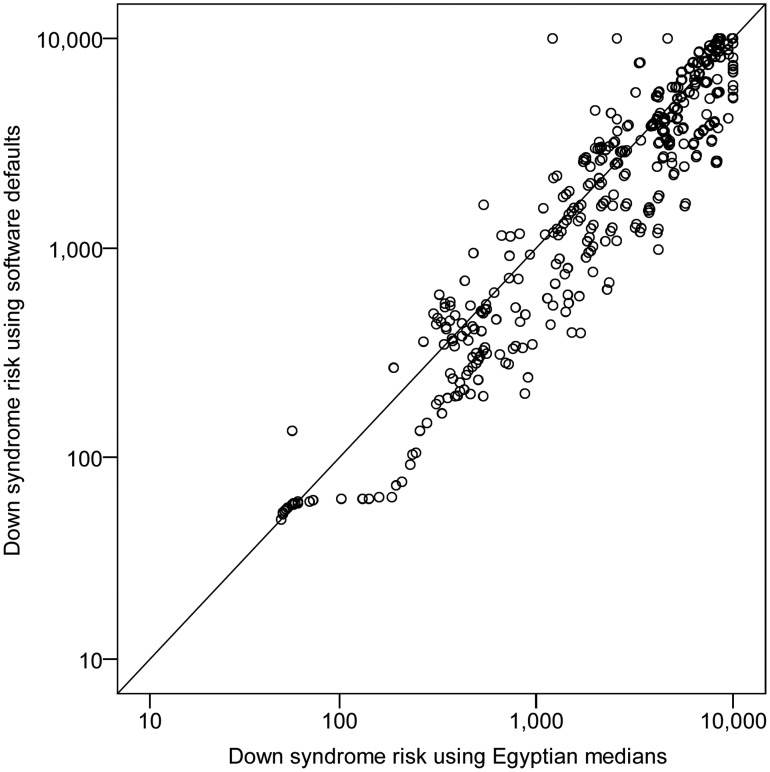
Comparison between risks (1:n) generated using Egypt-specific medians and software default medians.

Using the Egyptian-specific medians and weight correction factors; the DS risk for 45 out of the 668 screened women exceeded the risk cutoff adopted (1∶270 at sampling time). This figure equates to an initial positive rate (IPR) of 6.7%. When software default medians (European-based) were used, 76 cases were risky (IPR = 11.4%); including the 45 women identified risky when using Egyptian medians and extra 31 considered non-risky. In general, 67.7% of women were considered less risky when calculations were based on Egyptian medians, particularly in weeks 17 to 20. Figure 2 displays the general trend for overestimation of DS risk when default medians were blindly accepted.

Both prior (based on age) and posterior (based on Egyptian medians) risks were concordant (risk >1∶270) in 596 (89.2%) of females and were <1∶270 in 10 (1.5%) of them. The risks were discrepant in the remaining 62 (9.3%) participants; 27 (4.0%) were risky based on their ages only but turned-out to be non-risky after introducing biochemical markers into risk calculation, and 35 (5.3%) had an opposite scenario.

An error of at least 1 week was found in 8.5% of the studied females when last menstrual period was used for gestational dating. In half of them, the difference was 2 weeks or more. Such dating method caused doubling of initial positive rate from 6.7% to 13.9% compared to sonographic dating.

## Discussion

Successful, wide-spread implementation of a national screening programme for DS in Egypt is hampered by a number of hurdles. Developing a set of median values for each biochemical marker in every week of gestation comes on top of the list. Reliance on medians originating from other communities or accepting software defaults undermine the philosophy of such a population-specific programme and is postulated to be the primary cause for poor performance in our country. Other obstacles exist and need to be rectified as well. The current study was planned to address these issues aiming at promulgating a screening policy worthwhile adoption in Egypt. The study was conducted on 668 Egyptian females making sure that at least a hundred participants were included in each week between 15 and 20 as has been recommended [17]. The American College of Medical Genetics and Genomics, *(ACMG)* technical standards and guideline for DS screening state that ideally 100 samples for each gestational week from 15 through 20 should be used to calculate medians [18]. Definition of gestational age was consistently based on measurement of fetal biparietal diameter to circumvent errors associated with last menstrual period calculations. A difference of at least one week was found in 57 (8.5%) females, 14 of them were 2–3 weeks and 15 were 3–5 weeks. This finding underscores the importance of objective gestational dating using sonar [19], [20]; otherwise risk calculation would be erroneously based on analyte medians that belong to gestational ages 2–5 weeks away. Should this error be neglected in the current study, the initial positive rate was found to jump to 13.9%; more than double what sonographic age calculations furnished. It was calculated that an error of 3–4 days in gestational age can approximately double (or halve) the expected DS risk [17].

Median values for AFP, CG and uE3 were calculated for each week of the 2^nd^ trimester. Appropriate weighted regression analysis was used to fit the observed data to the GA. These equations are used to predict “smoothened” medians for each week of gestation, minimizing the effect of outliers and giving less weight to weeks with smaller number of participants. When these medians were compared to other geographical regions (Canada, UK, Germany, Belgium and Korea), they showed similar pattern of variation with GA (Figure 1). AFP increased by a constant proportion of 11.7% per week (11.3%–23.5% in the other regions) and uE3 increased by 28.4% (compared to 14.7%–35.7% elsewhere). CG, on the other hand, showed least association with GA, decreasing by 8.0% to 8.8%/week (8.0% to 19.6% in other countries). Errors in GA dating will, then, have its greatest impact on uE3. On a week to week basis, Egyptian AFP medians were second highest among other geographical regions; following Korean medians. Oestriol was lowest all through, particularly when compared to Korean medians that were higher by a factor of 1.6 to 2.1. CG occupied an intermediate position between Korea, Canada and UK (below them) and Belgium and Germany (above both). Interestingly, CG curve showed the least steep decline with GA compared to the five regions. The observed difference in medians is not purely racial, because the quoted measurements were made on Beckman Coulter Access rather than Siemens Immulite used in the current study. Both techniques, however, are immuno-chemiluminescence-based. Asian women who were reported to have the highest AFP, CG and uE3 [21], [22], are still leading even when compared to Egyptians.

Another potential source of error in DS risk calculation is the adjustment for maternal weight. Weight distribution in our community is different from that in Western, black or Asian communities, and needs to be considered when adjustments to MoMs are to be made. Three formulas were derived and served to adjust measured MoMs to a population median maternal weight of 78.2 kg (compared to 60 kg in a Caucasian-based study [23] and 57.2 kg in a Korean study) [15]. A difference in median weight as small as 2.5 kg was recommended to warrant formula modification to minimize errors in risk calculation [24]. This recommendation highlights the importance of using population-tailored equations. Neveux’s linear reciprocal model better fitted weight distribution in our community than the log-linear model recommended by Reynolds et al [24].

Due to the small number of diabetics (only 11 women) and absence of smoking history in the 668 participants, default software correction factors for these potential confounders were accepted. Multiple pregnancies were excluded from the study to avoid “pseudo-risk” calculations. Other suggested covariates (as parity, in vitro fertilization, intra-uterine insemination, analyte concentrations in a previous pregnancy or sex of the fetus) were beyond the scope of the current study. The improvement in screening that can be achieved by incorporating additional clinical factors needs to be balanced against the practical realities involved with the data collection [17].

In addition to the above statistical findings, a number of conventions need to be agreed upon by policy makers in order to harmonize results and unify terms across the whole country. The first convention is the time at which risk is to be calculated and expressed; whether mid-trimester (i.e., at sampling-time) or term-risk. The former choice was adopted in the current study. Another decision is the choice of DS risk cut-off that warrants invasive diagnostic procedures. A risk cut-off was set at 1∶270. It is equivalent to the risk of a 35-year old woman in the absence of serum screening. The risk cut-off of a screening test should be set specifically to each country in consideration of the cost and safety of invasive diagnostic procedures, the prevalence of DS, and the age distribution of pregnant women [15].

The appropriateness of findings and conventions recommended in the current study was assessed by epidemiological monitoring [25], [26]. Our target was to keep the percentage of women considered screen positive to a figure less than 7%. This figure, often termed initial positive rate (IPR) reached 6.7% in our study; well below our target. In many US laboratories using the same risk cut-off, same test-combination (triple test) and same method of gestational age dating (sonar); IPR was reportedly 6.5% [27].

Another statistic calculated was the grand MoM for each of the three measured parameters. It is the median of all 668 MoMs of each parameter; without regard for the gestational week. The target was to keep the 3 grand MoMs as close to unity as possible. All three MoMs were exactly 1.00.

It’s believed that a similar study has not been done in Egypt, nor in many countries in the Arab region and North Africa, and is hoped that implementation of the above medians, correction factors and conventions will pave the way to a successful national DS screening programme. Considering the ethnic similarities between most of the Arab women (Arab panethnicity), such recommendations can even extend to other countries in the region and would surely be a better option for some of them than accepting literature-based recommendations or software defaults.

## Supporting Information

Data S1 **Raw data.**
(XLSX)
